# Characterization of azithromycin-resistant *Shigella flexneri* serotype 2a isolates using whole genome sequencing in Ontario from 2016 to 2018

**DOI:** 10.1128/spectrum.00706-24

**Published:** 2024-09-09

**Authors:** Alefiya Neemuchwala, Karen Johnson, Kirby Cronin, Sandra Zittermann, Analyn Peralta, Vanessa G. Allen, Samir N. Patel

**Affiliations:** 1 Ontario Agency for Health Protection and Promotion (Public Health Ontario), Toronto, Ontario, Canada; 2 University of Toronto, Toronto, Ontario, Canada; University of California San Diego, La Jolla, California, USA

**Keywords:** *Shigella flexneri *serotype 2a, azithromycin-resistant, *mph*(A), *erm*B, travel

## Abstract

Azithromycin-resistant shigellosis is increasing globally. This retrospective analysis of *Shigella flexneri* serotype 2a isolates from 2016 to 2018 in Ontario found nearly half were azithromycin (47.7%, 72/151) and ciprofloxacin (50.7%, 77/152) resistant. Moreover, 34.7% (25/72) of azithromycin-resistant isolates were also ciprofloxacin-resistant. Four isolates were ceftriaxone-resistant, although all azithromycin-resistant isolates were ceftriaxone-susceptible. Overall, 83.6% (127/152) of all *S. flexneri* 2a isolates were recovered from males and 97.2% (70/72) of the azithromycin-resistant cases were males. Among the azithromycin-resistant cases, some (8/72) reported international travel. Phylogenetic analysis of azithromycin-resistant isolates revealed two large male-dominated clusters, and one cluster may have been due to importation of resistant strain. Comparison of plasmids isolated from the clusters in Ontario revealed the presence of incFII plasmid with high percentage of similarity to plasmids present in global outbreaks affecting mostly males including men who have sex with men (MSM). These two large azithromycin-resistant clusters are suggestive of an outbreak among MSM, though disease exposure or sexual orientation of patients was unknown. The presence of plasmid-borne azithromycin resistance in ciprofloxacin-resistant isolates is a public health concern. Antimicrobial surveillance is important for patient management, understanding the spread of novel resistance types in local communities which sometimes is introduced by travel. We found ongoing multidrug-resistant outbreaks spanning multiple years affecting males. Reduction of future outbreaks in high-risk communities like MSM requires consorted information flow between laboratory, public health, and physicians. We impart genomic and antimicrobial characteristics of multidrug *S. flexneri* 2a which may serve as reference by clinicians and public health.

**IMPORTANCE:**

Oral ciprofloxacin and azithromycin are generally considered as the first-line therapy of shigellosis. Here, we report the emergence and transmission of azithromycin and ciprofloxacin-resistant *S. flexneri* serotype 2a among male adults in Ontario during 2016–2018. The percentage of azithromycin and ciprofloxacin resistance among *S. flexneri* 2a is higher compared to previous reports from Canada and United States. Here, we show the genetic basis of the antimicrobial resistance among these unique groups of *S. flexneri* 2a isolates. We describe a domestically acquired azithromycin-resistant and ciprofloxacin-resistant *S. flexneri* 2a lineage in Ontario. Combining whole-genome sequencing (WGS) data with travel-associated data helped in understanding dissemination and transmission. We employed WGS, which not only helped us in understanding the genetic-relationship between isolates but also mine information regarding plasmids. In the future, linking WGS, travel-related data, and clinical data can provide enhanced contact tracing and improve public-health management.

## OBSERVATION

Shigellosis caused by *Shigella* spp. is typically treated with ciprofloxacin or azithromycin (1). In the last decade, azithromycin-resistant *Shigella flexneri* has caused multiple outbreaks among men who have sex with men (MSM) in Canada, Australia, the United States of America (USA), and the United Kingdom (UK) (2–8). In these outbreaks, the spread of azithromycin resistance was primarily due to a plasmid-borne *mph* gene (2–8).

Here, we present the genomic epidemiology of azithromycin-resistant *S. flexneri* 2a received at the Public Health Ontario (PHO) laboratory in Ontario, Canada, from January 1, 2016 to December 31, 2018. We investigated mechanisms of azithromycin resistance and linked genomic data to epidemiologic data, to understand prevalence among men and if travel was associated with azithromycin resistance in Ontario.

## THE STUDY

As the provincial reference laboratory, the PHO laboratory confirms the identification of species, serotyping, and susceptibility of *Shigella* spp. From January 1, 2016 to December 31, 2018, PHO laboratory received 152 *S*. *flexneri* 2a isolates, of which 83.6% were recovered from males (127/152, Fisher’s exact test *P* < 0.001) (Table 1). In 3 years, the percentage of isolates from males increased from 68.8% in 2016 to 92.0% in 2018 (Fisher’s exact test *P* < 0.001). A disparity in percentage distribution across age groups and sex was noted as 78.7% of males (100/127) were 20–59 years; in contrast, 56.0% of females (14/25) were aged 20–59 years. Although 68.3% (28/41) of foreign travelers were males, only 22.0% (28/127) of males were returning-travelers compared to 52% (13/25) of females.

**TABLE 1:** Characteristics of antimicrobial resistance in *Shigella flexneri* serotype 2a isolates in Ontario during 2016–2018^*d*^^,^^*e*^^,^^*f*^

			All cases	AZM^R^	CIP^R^	CRO^R^	SXT^R^	AMP^R^	CIP^IR^
*S. flexneri* serotype 2 a (all)	All	Total	152	72/151^*a*^ (47.7%)	77/152 (50.7%)	4/152 (2.6%)	100/152 (65.8%)	143/151^*a*^ (94.7%)	5/152 (3.3%)
	Gender	Male	127 (83.6%)	70 (97.2%)	61 (79.2%)	2 (50.0%)	84 (84.0%)	120 (83.9%)	4 (80.0%)
	Age-group (yrs)^*b*^	0–4 years	9 (5.9%)	0	5 (6.5%)	0	7 (7.0%)	8 (5.6%)	0
		5–19 yrs	9 (5.9%)	2 (2.8%)	6 (7.8%)	0	5 (5.0%)	9 (6.3%)	2 (40.0%)
		20–59 yrs	114 (75.0%)	61 (84.7%)	54 (70.1 %)	3 (75.0%)	73 (73.0%)	108 (75.5%)	3 (60.0%)
		>=60 yrs	19 (12.5%)	8 (11.1%)	12 (15.6%)	1 (25.0%)	14 (14.0%)	17 (11.9%)	0
	location	GTA	105 (69.1%)	52 (72.2%)	45 (58.4%)	2 (50.0%)	66 (66.0%)	97 (67.8%)	5 (100.0%)
		Ottawa	21 (13.8%)	10 (13.9%)	19 (24.7%)	0	18 (18.0%)	19 (13.3%)	0
	International Travel	(All regions)^*c*^	41 (27.0%)	8 (11.1%)	24 (31.2%)	1 (25.0%)	19 (19.0%)	36 (25.2%)	2 (40.0%)
		Africa	1 (2.5%)	1 (12.5%)	0	0	1 (5.3%)	1 (2.8%)	1 (50.0%)
		Asia	6 (15.0%)	1 (12.5%)	1 (4.2%)	0	3 (15.8%)	6 (16.7%)	0
		Central America	10 (25.0%)	0	6 (25.0%)	0	5 (26.3%)	8 (22.2%)	0
		Europe	3 (7.5%)	3 (37.5%)	1 (4.2%)	0	3 (15.8%)	3 (8.3%)	0
		South America	1 (2.5%)	0	1 (4.2%)	0	0	1 (2.8%)	0
		South Asia	15 (36.6%)	1 (12.5%)	14 (58.3%)	1 (100.0%)	6 (31.6%)	14 (38.9%)	1 (50.0%)
		United States	1 (2.5%)	1 (12.5%)	0	0	1 (5.3%)	1 (2.8%)	0
		The Caribbean	4 (10.0%)	1 (12.5%)	1 (4.2%)	0	0	2 (5.6%)	0
AZM^R^ *S. flexneri* 2a	All	all	N/A	72	25/72 (34.7%)	0	60/72 (83.3%)	72/72 (100.0%)	1/72 (1.4%)
	Gender	Male	N/A	N/A	24 (96.0%)	0	59 (98.3%)	70 (97.2%)	0
	Age-group (years)	0–4 years	N/A	N/A	0	0	0	0	0
		5–19 years	N/A	N/A	1 (4.0%)	0	1 (1.7%)	2 (2.8%)	0
		20–59 years	N/A	N/A	19 (76.0%)	0	50 (83.3%)	61 (84.7%)	1 (100.0%)
		>= 60 years	N/A	N/A	5 (20.0%)	0	8 (13.3%)	8 (11.1%)	0
	Location	GTA	N/A	N/A	14 (56.0%)	0	44 (73.3%)	52 (72.2%)	1 (100.0%)
	Travel	International travel	N/A	N/A	3 (12.0%)	0	6 (10.0%)	8 (11.1%)	1 (100.0%)

^*a*^ Represents the total number of isolates whose susceptibility was known for the antimicrobial tested.

^*b*^ Age was unknown for one.

^*c*^ Travel information could not be linked for three isolates received at the laboratory.

^*d*^ The total number of resistant (R) and intermediate (IR) isolates with the percentage (in brackets) is indicated for each antimicrobial. Also shown is the distribution of azithromycin-resistant isolates that are co-resistant to other antimicrobial.

^*e*^ ±For cases reporting international travel, travelers reported countries or continents. For reporting, these were aggregated as continents. For the Americas, we subclassified regions as South America, Central America, United States, and Caribbean countries. Countries from Asia were further subclassified into South Asia and Southeast Asia. In the table, the count for Asia indicates other Asian countries that are not in South Asia or Southeast Asia.

^*f*^ Azithromycin (AZM), ciprofloxacin (CIP), ceftriaxone (CRO), trimethoprim-sulfamethoxazole (SXT), ampicillin (AMP), GTA- Greater Toronto Area, N/A Not Applicable.

We found 47.7% (72/151) of *S. flexneri* 2a in Ontario were azithromycin-resistant, 50.7% (77/152) were ciprofloxacin-resistant, 65.8% (100/152) were trimethoprim-sulfamethoxazole-resistant, 94.7% (143/151) were ampicillin-resistant and 2.6% (4/152) were ceftriaxone-resistant (Table 1). The percentage of azithromycin-resistant *S. flexneri* 2a isolates increased from 35.4% (17/48) in 2016 to 56.0% (28/50) in 2018. Furthermore, 34.7% (25/72) and 83.3% (60/72) of azithromycin-resistant isolates were resistant to ciprofloxacin and trimethoprim-sulfamethoxazole, respectively. Among azithromycin-resistant isolates, 30.6% of isolates (22/72) were resistant to both ciprofloxacin and trimethoprim-sulfamethoxazole. Most azithromycin-resistant isolates (97.2%) were recovered from males [(70/72); Fisher’s exact test *P* < 0.001]. Azithromycin-resistant cases were primarily found in the Greater Toronto Area (GTA) [72.2% (52/72)] and Ottawa [13.9% (10/72)]. All azithromycin-resistant *S. flexneri* 2a isolates, except two, were recovered from adults 20 years or older. Eight azithromycin-resistant cases (11.1%, 8/72) reported foreign travel, of which seven (87.5%, 7/8) were male.

Whole-genome sequencing (WGS) was performed retrospectively on all available azithromycin-resistant isolates (*n* = 68) and randomly chosen azithromycin-susceptible isolates (*n* = 11), using Nextera XT paired-end kits on the Illumina platform (Table S1). Sequencing and analysis were done similarly as described earlier (5) and detailed in Supplementary methods.

**Fig 1:**
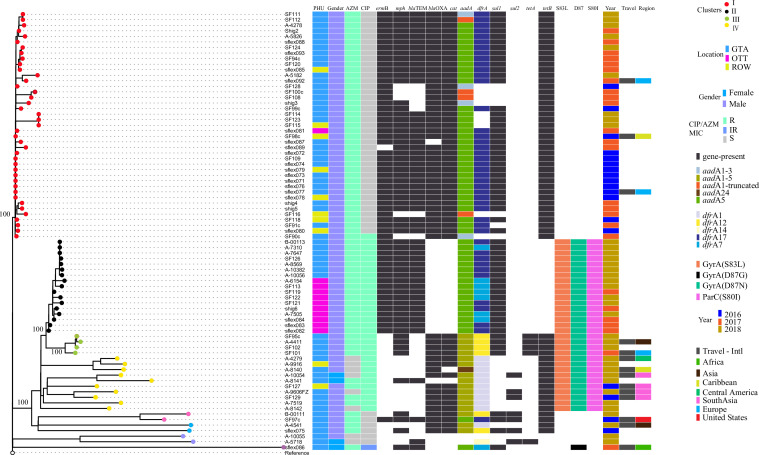
Maximum likelihood tree (ML-tree) showing the phylogeny (unrooted tree) of azithromycin-resistant *Shigella flexneri* 2a circulating in Ontario during 2016–2018. The various antimicrobial-resistant genes are illustrated as genes present (dark grey blocks) or absent (no coloring) identified using ResFinder and SRST2 are shown. Also shown is the susceptibility to antimicrobials azithromycin (AZM) and ciprofloxacin (CIP) which are colored according to the legend as resistant (R), susceptible (S), or intermediate-resistant (IR). Also included are the patient’s gender, travel destination (if international travel was reported) and location. Locations were grouped as Greater Toronto Area (GTA), Ottawa region (OTT) and rest of Ontario (ROW). Isolates are colored per cluster and the two main clusters are denoted with red and black circles as clusters I and II. Clusters III and IV were represented with green and yellow circles. Other non-clustering isolates belonging to different lineages were shown with pink, light blue, cyan-blue, and purple colours. Isolates that were recovered from patients reporting international travel status are dark grey and destination colored according to the inset key. ML tree was generated using 1646 coreSNPS identified using an in-house pipeline and after removing regions of recombination, insertion sequences, genomic islands, and phages against reference *S. flexneri* 2a-2457T (GenBank AE014073.1) using IQ-TREE with 1000 ultraFAST bootstraps.

Phylogenetic analysis against *S. flexneri* 2457T (GenBank AE014073.1), identified distinct azithromycin-resistant clonal populations in Ontario (Fig. 1). A monophyletic clonal population (cluster I) circulating between 2016 and 2018 accounted for 60.3% (41/68) of azithromycin-resistant cases (Fig. 1; Table 2). Isolates among cluster I differed by 0–35 single nucleotide polymorphisms (∆SNP). Cluster I isolates were exclusively recovered from males and most were GTA residents [78.0% (32/41)]. The first case from cluster I in February 2016 reported international travel to the Dominican Republic and the corresponding isolate differed by 0–4 ∆SNP from other clustering isolates received from February 2016 to August 2016, suggesting international travel as the source of the clone followed by local circulation.

**TABLE 2:** Characteristics of the two major *Shigella flexneri* serotype 2a clusters identified using whole-genome sequencing (WGS) that caused 80.6% (58/72) of azithromycin-resistant cases in Ontario

	Cluster I	Cluster II
∆SNP	0–35^*a*^	0–14
Total cases	41	17
Males	41	17
Age-range		
0–4 years	0	0
5–19 years	1	0
20–59 years	36	12
≥60 years	3	5
Location		
GTA	32	7
Ottawa	1	9
International travel		
No. of cases reporting international travel	3	0
International travel region traveled (*n*)	Europe (2), The Caribbean (1)	N/A
Antimicrobial resistance		
No. of AZM^R^	41	17
No. of CIP^R^	1	17

^*a*^ All isolates except four had 0–10 ∆SNP.

**Fig 2:**
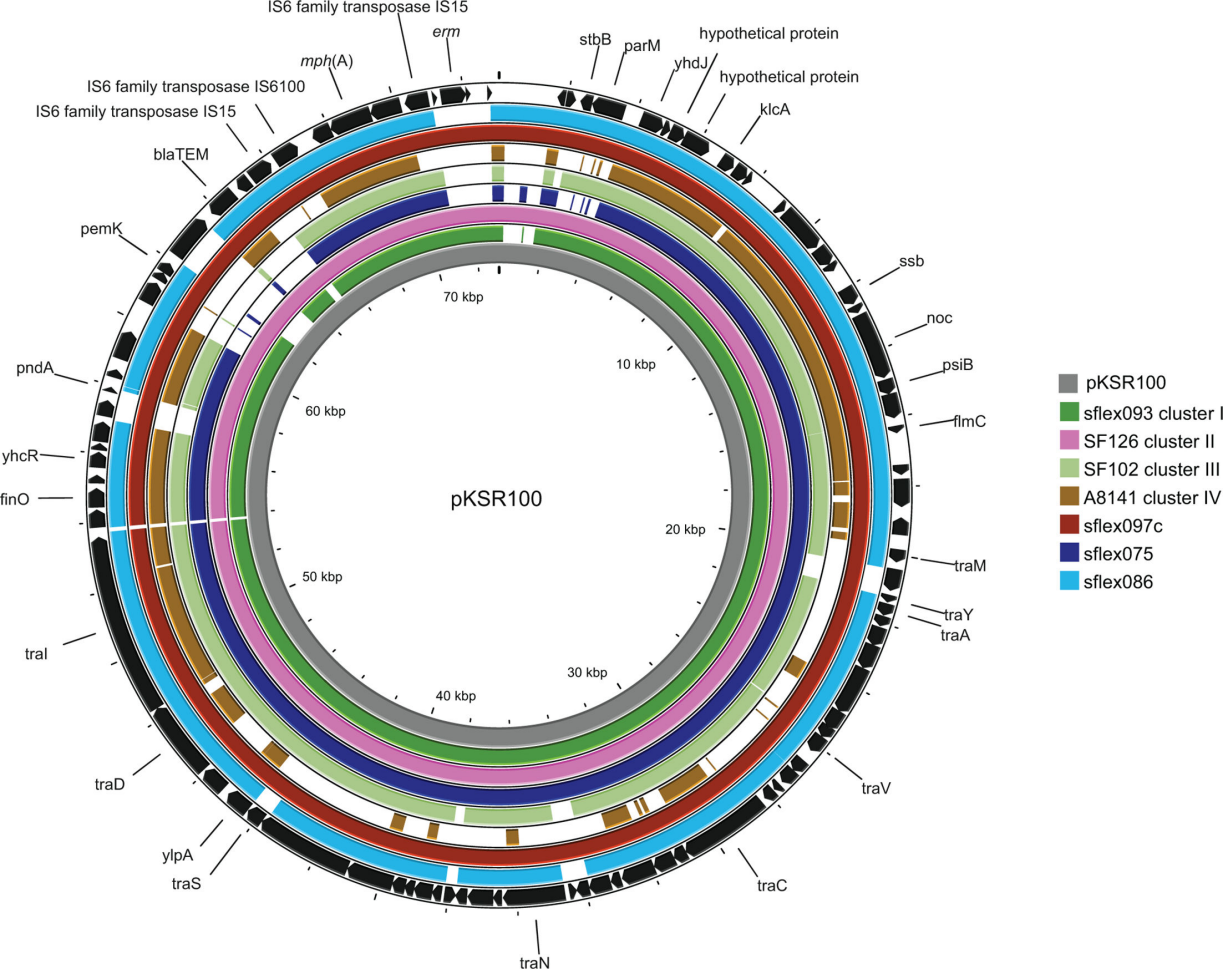
Figure illustrating the homology of *mph*(A) gene-bearing plasmids present in Ontario to pKSR100 (LN624486) using Proksee. The gray inner-most ring backbone represents pKSR100, followed by Ontario isolates from each cluster (see inset legend).

The second azithromycin-resistant group (cluster II, *n* = 17) appeared in 2017 and the isolates recovered in 2017 were mainly from Ottawa (*n* = 5/6, 1–5 ∆SNP) with later spread to Toronto in 2018 (*n* = 7, 0–5 ∆SNP). All cluster II isolates were co-resistant to ciprofloxacin, bearing triple mutations in the quinolone resistance determining region [(QRDR), GyrA (S83L and D87N), and ParC (S80I)]. Azithromycin-resistant isolates from both clusters contained an incFII plasmid with significant homology to the pKSR100 plasmid (LN624486) (Fig. 2). This plasmid had both *mph* and *erm*B genes though five cluster I isolates only had the *erm*B gene. Additional resistance genes (*bla*_TEM-1B_, *aad*A5, *dfr*A17, and *sul*1) were present in 70.7% (29/41) cluster I and 58.8% (10/17) cluster II isolates.

Four other isolates co-resistant to azithromycin and ciprofloxacin with only the *mph*A gene and having triple QRDR mutations grouped together [cluster III (2–22∆SNP), Fig. 1 and 2], two were travel-associated. A fourth group (*n* = 10, cluster IV) consisted mostly of azithromycin-susceptible isolates (80%, 8/10), but all were ciprofloxacin-resistant and 60.0% were travel-associated (*n* = 6/10). Among the two azithromycin-resistant cluster IV isolates, one had a plasmid-borne *mph* gene and no known genetic resistance marker was found in the other (MIC >128 mg/L).

Four other non-clustering travel-related azithromycin-resistant isolates had the *mph* gene and one among them, was also intermediate resistant to ciprofloxacin with a GyrA (D87G) genotype. Another azithromycin-resistant isolate (B-00111) had the *mef*E gene as well as the *mph*A gene. The MIC of azithromycin was >32 mg/L for isolates with mph(A) only or with the *erm*B gene. According to the 2021 azithromycin breakpoints, an MIC of 16 mg/L indicates intermediate resistance and previously it was interpreted as non-wild-type (NWT) with epidemiological cutoff values (9). Among isolates lacking *mph* but having the *erm*B gene only, the MIC of azithromycin was 16 mg/L which concurs previous reports (3, 10–12).

## DISCUSSION

Our analysis revealed two distinct large clonal populations responsible for 80.6% (58/72) of azithromycin-resistant isolates found in Ontario. Azithromycin resistance was due to the presence of the *mph*A and/or *erm*B gene on plasmids that are similar to pKSR100 plasmids associated with MSM outbreaks elsewhere (1–8). Azithromycin-resistant *S. flexneri* co-resistant to ciprofloxacin, ampicillin, and trimethoprim-sulfamethoxazole were reported earlier among MSM in Canada, US, UK, and Australia (3–7). In our study, azithromycin-resistant cases were primarily in males suggesting that they may be part of a spread related to MSM; however, our data lacks disease exposure, contact history, or sexual history of patients. Historically, the risk of acquiring *S. flexneri* from travel to other developed nations is presumed to be low (2, 3). Although the percentage of travel-related azithromycin-resistant cases is low, WGS analysis demonstrates similarities in strains found in Ontario with other developed nations.

In conclusion, using WGS, we found both travel-related and domestically circulating azithromycin-resistant clusters of public health interest. Among the isolates sequenced in Ontario, at least four ciprofloxacin- and azithromycin-resistant clusters were present. Spread of ciprofloxacin-non-susceptible and azithromycin-resistant *S. flexneri* is a major public health concern as treatment failures among immunocompromised, and malnourished, or elderly patients have been observed (13, 14). We observed that 47.7% of isolates in Ontario were azithromycin-resistant which is similar to reports from the UK during 2015–2019 (15). Both the total proportion (48.3%, 152/315) and travel-associated (27.0%, 41/315) *S. flexneri* 2a cases was higher than serotype 1b in Ontario (5). Though the proportion of females travelers in Ontario was similar for serotypes 1b (54.5%) and 2a (52%), travel was reported more among men afflicted with *S. flexneri* 2a (22%) than *S. flexneri* 1b (11.3%) (5), showing different risk-factors and sources exist at serotype level. Some men in azithromycin-resistant clade I in Ontario are returning travelers from the Caribbean and Europe. Incidentally, *S. flexneri* isolates in MSM clusters in the UK during 2015–2017 had few returning-travelers from Europe or the Caribbean, that report a similar phenotype as cluster I (15). While high incidence among men is mostly MSM-associated (1, 2), 28% of MSM in a study reported no recent sexual contact, suggesting non-sexual transmission (15). As disease-exposure was unknown to us, we are unable to clearly link the high incidence among men to sexual transmission. However, the potential for non-sexual transmission requires additional study including social-behavioral or hygiene-related risk factors. Educating high risk-populations of how shigellosis is transmitted may reduce outbreaks. Another alternate reason explaining the high incidence rate could be delayed symptom-onset as suggested by authors (15), which poses a challenge for contact tracing as most public-health outbreak shigellosis investigation tool consider an incubation period of 1–4 days for *S. flexneri*. Many consider the high incidence of azithromycin-resistant shigellosis among MSM may be due to the selective pressure of azithromycin, which is commonly prescribed to treat other STIs (1, 2).

Contact tracing that links recent travel or contact with returning travelers may aid to reduce the further spread of highly antimicrobialresistant *Shigella*. Additional integration of risk factors such as exposure through sexual activity with WGS and laboratory findings will aid infection control and outbreak management. Ongoing studies using WGS are needed to understand the global distribution of lineages, ∆SNP and local persistence of clones and the acquisition of resistance. Our findings raise awareness among clinicians and antimicrobial stewards of the potential clinical failure of empirical antimicrobial agents used in shigellosis.

## Data Availability

The Fastq files of *S. flexneri* 2a from Ontario included in this study are submitted to Bioproject PRJNA1069659 in the National Centre Biotechnology Information (NCBI) database. Please refer to Supplementary Table S1 for more information.

## SUPPLEMENTAL MATERIAL

Supplemental material spectrum.00706-24-s0001.docx Methods; Supplementary Table S1.

TABLE S1 spectrum.00706-24-s0002.xlsx Characteristics of the Shigella flexneri 2a isolates in Ontario 2016-2018.

ASM does not own the copyrights to Supplemental Material that may be linked to, or accessed through, an article. The authors have granted ASM a non-exclusive, world-wide license to publish the Supplemental Material files. Please contact the corresponding author directly for reuse.
