# Supplementary material for: Characterization of azithromycin-resistant Shigella flexneri serotype 2a isolates using whole genome sequencing in Ontario from 2016 to 2018
Source: Microbiol Spectr. 2024 Sep 9;12(10):e00706-24. doi: 10.1128/spectrum.00706-24 (PMC11448384; doi:10.1128/spectrum.00706-24)
Supplement: Supplemental material — Methods; Supplementary Table S1. [file spectrum.00706-24-s0001.docx]

**Supplementary Material**

**Methods**

**Data Sources**

The province of Ontario in Canada has 34 Public Health Units (PHU) who notify shigellosis cases by reporting them into the Integrated Public Health Information System (iPHIS). For our study, records of laboratory confirmed shigellosis cases received at Public Health Ontario Laboratory from January 1, 2016 to December 31, 2018, were extracted (including age, sex, public health unit and travel-status if known). We grouped location for isolates recovered from five PHUs (Toronto, Peel, Halton, York and Durham) as Greater Toronto Area (GTA) for our study. Travel information was classified as ‘international travel’ if travel outside of Canada was reported within four days of symptom onset or was termed as ‘domestic travel’ if travel outside of province but within Canada travel was reported. This information was retrieved from iPHIS.

**Ethical Approval**

The data from iPHIS was linked with Public Health Ontario’s (PHO) laboratory data as part of routine surveillance initiative. All personal identifiers linked to laboratory specimen numbers were removed. Whole genome sequencing (WGS) was done on de-identified specimens as part of surveillance investigation, and therefore did not require review by ethical review board.

**Data Management**

Any duplicated isolates received within 15 days from the same patient at PHO’s Laboratories were removed from analysis. There were 159 isolates received at the PHO laboratory from January 1, 2016 to December 31, 2018. After removing duplicates, 152 isolates were included in our study. Of these data, 149 isolates were linked with case records present in the iPHIS database.

**Bacterial isolates, Susceptibility Testing and Whole Genome Sequencing**

On receipt, isolates were serotyped using in-house protocols consisting of biochemical tests and slide agglutination assays against specific antisera which were previously published (1-3). Susceptibility testing was performed against a panel of antibiotics for all isolates using the agar dilution method as per Clinical laboratory standards Institute (CLSI) (4). For the purpose of our study we analysed susceptibility results of ampicillin, ciprofloxacin, azithromycin, ceftriaxone and trimethoprim-sulfamethoxazole.

Susceptibility testing of azithromycin and ceftriaxone were retrospectively performed by testing concentrations ranging from 2 to 128mg/L for azithromycin and 1 to 8mg/L for ceftriaxone by agar dilution method (4). MICs of azithromycin and ceftriaxone for some isolates were determined using the E-test (Alere, Stittsville, Ontario) according to manufacturer’s instruction and CLSI protocols. Susceptibility was interpreted as outlined in the 28^th^ edition of guidelines from Clinical laboratory standards Institute (CLSI) (5).

WGS was performed retrospectively on select isolates to characterise the azithromycin-resistant *S. flexneri* 2a isolates (n=79, azithromycin-resistant=68). Genomic DNA was extracted using Qiagen DNA extraction kit (Qiagen Inc.) and WGS was performed on Illumina Miseq (Illumina Inc.). Libraries were prepared as per manufacturer’s instruction using Nextera XT paired-end kits and sequenced on the Miseq employing either (151+151) v2 chemistry or (301+301) v3 chemistry kits (Illumina Inc.).

**Bio Informatic Analysis**

The quality of sequences was assessed using FASTQC and Confindr (6). Phylogenetic analysis was performed by calling SNPs against a previously closed reference genome *S. flexneri* 2a 2457T (GenBank accession no. AE014073.1) using an in-house pipeline that employs SMALT, FreeBayes and SAMtools to generate high quality SNPs. Variants in regions that identified as prophages, genomic islands, repetitive regions and recombination regions as identified by PHASTER (7), Island Viewer 4 (8) and Gubbins (9) were excluded to yield 1646 variant sites and a maximum likelihood tree (ML-Tree) was generated using IQ-TREE version 1.6.2(10). SNP distance (∆SNP) between isolates was calculated using SNP-dists (https://github.com/tseemann/snp-dists) (11).

Antimicrobial resistance genes were analysed using the SRST2 (v0.1.8) with ResFinder (v3.0) as database and RGI (v4.2) (12-14). The ML tree with metadata was visualised using ggtree (15). Whole genome assemblies were obtained using SPAdes (v3.9) (16). We used multiple methods to establish presence of plasmids and map homology to pKSR100. These included mapping reads against pKSR100 using BLASTn and by using MOB-suite (v.1.4) (17-18). Homology of Ontario plasmids were illustrated using Proksee (19) by performing BLAST against pKSR100 (GenBank LN624486).

**Statistical Analysis**

We analysed categorical variables using the Fisher’s exact test in R (4.1) and consider P values of < 0.001 as significant.

**References**

1. Ewing W.H. 1986. The genus *Shigella* p.135-172. In Edwards and Ewing's Identification

of *Enterobacteriaceae* 4th ed., Elsevier Science Publishing Co., New York.

1. Ewing W.H. 1971. Biochemical reactions of *Shigella*. DHEW Publication No. (H5M) 72-8081. Centers for Disease Control, Atlanta.
2. Murray, P. R.  *2011.* *Manual of Clinical Microbiology* 10th edn. (ASM Press, 2011).
3. Clinical Laboratory Standards Institute. 2016. Methods for Dilution Antimicrobial Susceptibility Tests for Bacteria that Grow Aerobically. Wayne, PA: Clinical and Laboratory Standards Institute.
4. Clinical Laboratory Standards Institute CLSI. 2018. Performance standards for antimicrobial susceptibility testing.28th ed.CLSI Supplement M100. Wayne,PA: Clinical and Laboratory Standards. 2018. CLSI.
5. Low, A.J., Koziol, A.G., Manninger, P.A., Blais, B. & Carrillo, C.D. 2019. ConFindr: rapid detection of intraspecies and cross-species contamination in bacterial whole-genome sequence data. PeerJ **7**, e6995; https://doi.org/10.7717/peerj.6995.
6. Arndt D, Marcu A, Liang Y, Wishart DS. 2017 Sep 25. PHAST, PHASTER and PHASTEST: Tools for finding prophage in bacterial genomes. Brief Bioinform.
7. Bertelli C, Laird MR, Williams KP, Simon Fraser University Research Computing G, Lau BY, Hoad G, Winsor GL, Brinkman FSL . 2017 Jul3. IslandViewer 4: expanded prediction of genomic islands for larger-scale datasets. Nucleic acids Res. 2017 Jul 3; 45(W1):W30-W5.
8. Croucher NJ, Page AJ, Connor TR, Delaney AJ, Keane JA, Bentley SD, Parkhill J, Harris SR. 2015 Feb 18. Rapid phylogenetic analysis of large samples of recombinant bacterial whole genome sequences using Gubbins. Nucleic acids Res.43(3):e15.
9. Nguyen LT, Schmidt HA, von Haeseler A, Minh BQ. 2015. IQ-TREE: a fast and effective stochastic algorithm for estimating maximum-likelihood phylogenies. MolBiol Evol. 32(1):268-74.
10. https://github.com/tseemann/snp-dists.
11. Zankari E, Hasman H, Cosentino S, Vestergard M, Rasmussen S, Lund O, Aarestrup FM, Larsen MV. 2012. Identification of acquired antimicrobial resistance genes. J Antimicrob Chemother. 67(11):2640-2644. doi:10.1093/jac/dks261
12. Inouye M, Dashnow H, Raven LA, Schultz MB, Pope BJ, Tomita T, Zobel J, Holt KE. 2014. SRST2: Rapid genomic surveillance for public health and hospital microbiology labs. Genome Med. 6(11):90.
13. Alcock BP, Raphenya AR, Lau TTY, Tsang KK, Bouchard M, Edalatmand A, et al. 2020. CARD 2020: antibiotic resistome surveillance with the comprehensive antibiotic resistance database. Nucleic acids research. 48(D1):D517-D25.
14. Yu G. 2020. Using ggtree to Visualize Data on Tree-Like Structures. Curr Protoc Bioinformatics. 69(1):e96. doi: 10.1002/cpbi.96.
15. Bankevich A, Nurk S, Antipov D, Gurevich AA, Dvorkin M, Kulikov AS, et al. 2012. SPAdes: a new genome assembly algorithm and its applications to single-cell sequencing. J Comput Biol. 19(5):455-77.
16. Altschul SF, Gish W, Miller W, Myers EW, Lipman DJ. 1990. Basic local alignment search tool. J Mol Biol. 215(3):403-10.
17. Robertson J, Nash JHE. 2018 Aug. MOB-suite: software tools for clustering, reconstruction and typing of plasmids from draft assemblies. Microb Genom. 4(8).
18. Grant  JR, Enns E, Marinier E, Mandal A, Herman EK, Chen CY, Graham M, Domeselaar GV, Stothard P. 2023. Proksee: in-depth characterization and visualization of bacterial genomes, Nucleic Acids Res. <https://doi.org/10.1093/nar/gkad326>.
